# Suppression of manganese superoxide dismutase augments sensitivity to radiation, hyperthermia and doxorubicin in colon cancer cell lines by inducing apoptosis

**DOI:** 10.1054/bjoc.2000.1367

**Published:** 2000-09-04

**Authors:** S Kuninaka, Y Ichinose, K Koja, Y Toh

**Affiliations:** Clinical Research Institute, Department of Chest Surgery, Gastroenterologic Surgery, National Kyushu Cancer Center, Notame 3-1-1, Minami-ku, Fukuoka 811-1395, Japan; Second Department of Surgery, School of Medicine, University of Ryukyus, Okinawa, Japan

**Keywords:** Mn-SOD, radiation, hyperthermia, sensitivity, apoptosis

## Abstract

Increased expression of manganese superoxide dismutase (Mn-SOD), one of the mitochondrial enzymes involved in the redox system, has been shown to diminish the cytotoxic effects of several anti-cancer modalities, including tumour necrosis factor-α, ionizing radiation, certain chemotherapeutic agents and hyperthermia. We asked if Mn-SOD is a potential target to augment the sensitivity of cancer cells to various anti-cancer treatments and for this we established stable Mn-SOD antisense RNA expressing cell clones from two human colon cancer cell lines, HCT116 (p53 wild-type) and DLD1 (p53 mutant-type). Suppression of Mn-SOD in HCT116 was accompanied by an increased sensitivity to radiation, hyperthermia and doxorubicin, as compared with findings in controls. The mitochondrial permeability transition, as measured by a decrease of the mitochondrial transmembrane potential was more intensely induced by radiation in HCT116 antisense clones than in the control, an event followed by a greater extent of DNA fragmentation. Apoptosis was also induced by hyperthermia more intensely in HCT116 antisense clones than in the control. On the other hand, DLD1 antisense clones did not exhibit any enhancement of sensitivity to any of these treatments. These data support the possibility that inhibition of Mn-SOD activity renders colon cancer cells with wild-type p53 susceptible to apoptosis induced by radiation, hyperthermia and selected anti-cancer drugs. Therefore, we suggest that Mn-SOD could be a target molecule to overcome the resistance to anti-cancer treatments in some colon cancer cells carrying wild-type p53. © 2000 Cancer Research Campaign


*British Journal of Cancer* (2000) **83**(7), 928–934
© 2000 Cancer Research Campaign
doi: 10.1054/ bjoc.2000.1367, available online at http://www.idealibrary.com on IDEAL®

### Suppression of manganese superoxide dismutase augments sensitivity to radiation, hyperthermia and doxorubicin in colon cancer cell lines by inducing apoptosis

**S Kuninaka[1], Y Ichinose[2], K Koja[4] and Y Toh[3]**

[1]Clinical Research Institute; [2]Department of Chest Surgery; [3]Gastroenterologic Surgery, National Kyushu Cancer Center, Notame 3-1-1, Minami-ku, Fukuoka 811-1395, Japan; [4]Second Department of Surgery, School of Medicine, University of Ryukyus, Okinawa, Japan

**Summary** Increased expression of manganese superoxide dismutase (Mn-SOD), one of the mitochondrial enzymes involved in the redox system, has been shown to diminish the cytotoxic effects of several anti-cancer modalities, including tumour necrosis factor-α, ionizing radiation, certain chemotherapeutic agents and hyperthermia. We asked if Mn-SOD is a potential target to augment the sensitivity of cancer cells to various anti-cancer treatments and for this we established stable Mn-SOD antisense RNA expressing cell clones from two human colon cancer cell lines, HCT116 (p53 wild-type) and DLD1 (p53 mutant-type). Suppression of Mn-SOD in HCT116 was accompanied by an increased sensitivity to radiation, hyperthermia and doxorubicin, as compared with findings in controls. The mitochondrial permeability transition, as measured by a decrease of the mitochondrial transmembrane potential was more intensely induced by radiation in HCT116 antisense clones than in the control, an event followed by a greater extent of DNA fragmentation. Apoptosis was also induced by hyperthermia more intensely in HCT116 antisense clones than in the control. On the other hand, DLD1 antisense clones did not exhibit any enhancement of sensitivity to any of these treatments. These data support the possibility that inhibition of Mn-SOD activity renders colon cancer cells with wild-type p53 susceptible to apoptosis induced by radiation, hyperthermia and selected anti-cancer drugs. Therefore, we suggest that Mn-SOD could be a target molecule to overcome the resistance to anti-cancer treatments in some colon cancer cells carrying wild-type p53. © 2000 Cancer Research Campaign

**Keywords**: Mn-SOD; radiation; hyperthermia; sensitivity; apoptosis

Superoxide dismutase (SOD) is an antioxidant enzyme involved in the defence systems against reactive oxygen species (ROS). There are three known SODs, including mitochondrial manganese SOD (Mn-SOD), intracellular copper, zinc SOD (Cu,Zn-SOD), which localizes at the cytoplasm and nucleus, and extracellular Cu,Zn-SOD. These SODs catalyse dismutation of two superoxide anions ($O_2^{\cdot-}$) into hydrogen peroxide, which is then catalysed to innocuous $O_2$ and $H_2O$ by glutathione peroxidase and catalase. Excessive ROSs provoke untoward events such as DNA damage, peroxidation of the lipid membrane and protein (Janssen et al, 1993). In addition, ROS are also thought to act as a potent mediator of apoptosis (Oberley and Buettner, 1979; Buttke and Sandstrom, 1994). Indeed, ionizing radiation, hyperthermia, some chemotherapeutic agents and cytokines elicit anti-tumour effects by generating a large amount of noxious radicals in the affected cells (Sinha and Mimnaugh, 1990; Yoshikawa et al, 1993; Wallace, 1999). The overexpression of Mn-SOD which dismutases $O_2^{\cdot-}$ radicals in the mitochondria makes the cancer cells more resistant to treatments in cases of malignant melanoma, breast and cervical cell carcinomas (Wong et al, 1989; Hirose et al, 1993; Li and Oberley, 1997).

*Received 15 February 2000*
*Revised 22 May 2000*
*Accepted 25 May 2000*

*Correspondence to:* Y Toh

The expression level of Mn-SOD is always altered in cancers (Oberley and Oberley, 1997) and it was reported that forced expression of Mn-SOD in various tumour cell lines converts malignant phenotypes to less malignant or benign ones (Church et al, 1993; Li et al, 1995; Zhong et al, 1996). These data support the concept that Mn-SOD may be a new type of tumour suppressor gene. On the other hand, increasing evidence challenging this concept has been reported; high levels of Mn-SOD expression were observed in malignant tumours such as central nervous system tumour, colorectal cancer and mesothelioma (Cobbs et al, 1996; Kinnula et al, 1996; Landriscina et al, 1996; Janssen et al, 1998). Some of these reports also revealed that the higher Mn-SOD expression correlates with a more advanced stage of the cancer or shortened survival time. We earlier examined the correlation between Mn-SOD mRNA expression levels and clinicopathological parameters in gastric and colorectal cancers and found that Mn-SOD mRNA was more highly expressed in the tumour as compared with the corresponding normal mucosa, and the expression level of Mn-SOD mRNA significantly correlated with the aggressiveness of the cancers (Toh et al, 2000).

Given that highly expressed Mn-SOD in colon cancers confers resistance to anti-cancer therapies, inhibition of endogenous Mn-SOD expression level or activity may be one strategy to overcome the resistance of these cancers. Thus, we attempted to clarify the significance of Mn-SOD in sensitivity of colon cancer cells to radiation, hyperthermia and chemotherapeutic agents and we investigated the potential of Mn-SOD to be a target for enhancing the effectiveness of anti-cancer therapies.

### MATERIALS AND METHODS

### Cell line

HCT116 and DLD1 colon adenocarcinoma cells were maintained in McCoy's 5A and RPMI 1640 medium supplemented with 10% heat-inactivated fetal bovine serum, respectively, in humidified 95% air and 5% $CO_2$.

### Construction of the Mn-SOD antisense RNA expression plasmid

A 216 bp Mn-SOD cDNA fragment, which encompasses the 5′ part of the cDNA including translation initiation codon, was synthesized by reverse transcription-polymerase chain reaction amplification (RT-PCR), using primers (5′-primer, 5′-catcagcg-gtaagccagcac-3′; 3′-primer, 5′-gttcaggttgttcacgtagg-3′) with the total RNA extracted from HCT116 colon cancer cell as a template and inserted into a eukaryotic expression vector pCR3.1 uni (Invitrogen, Carlsbad, CA, USA) in the reverse direction. The sequence of Mn-SOD fragment was confirmed by automated DNA sequencer (Applied Biosystems, Foster City, CA, USA).

### Establishment of stable clones expressing Mn-SOD antisense RNA

Transfection of the resultant expression plasmid or the vector only to HCT116 and DLD1 was performed using LipofectAMINE (Life Technologies Inc., Grand Island, NY, USA) according to the manufacturer's instruction. All transfectants were selected under the concentration of 400 μg $ml^{-1}$ neomycin (Life Technologies Inc.) for approximately 2 weeks and thereafter cloned by limiting dilution. The resultant stable transfectants were maintained under selection pressure (400 μg $ml^{-1}$ neomycin) during all experiments described below. Control transfectants carrying only the self-ligated pCR3.1 uni were cloned using the same procedure.

### Northern blotting

Total RNA was extracted from parental cell and transfectants using ISOGEN (Nippon Gene, Tokyo, Japan), a monophase solution with phenol and isothianate. The RNA was suspended in RNase-free water, and separated by electrophoresis through 18% formaldehyde, 1.5% agarose gel. The fractionated RNA was transferred onto a nylon membrane, and cross-linked with UV linker. The filter was hybridized with the $^{32}P$-labelled Mn-SOD cDNA probe or glyceraldehyde-3-phosphate dehydrogenase cDNA probe. After washing, autoradiogram was imaged and analysed with BAS system (Fuji Film Co Ltd, Tokyo, Japan).

### Mn-SOD activity assay and native gel assay

The Mn-SOD activity assay was performed according to Oberley and Spitz (1985). In brief, Mn-SOD activity of the sonicated homogenates of the cultured cells was assayed by competitive inhibition of $O_2^{\cdot -}$ generated by the xanthine–xanthine oxidase system to reduce nitroblue tetrazolium (NBT; Sigma, St. Louis, MO, USA) in the presence of sodium cyanide. The reduction rate of NBT was monitored spectrophotometrically at 560 nm. Differences in Mn-SOD activity between antisense and control clones were obtained by comparing the inhibitory effect of each sample on reducing NBT in the same amounts of protein. In some experiments, both Mn-SOD activity and Cu,Zn-SOD activity were also monitored using the native gel assay, as described (Beauchamp and Fridovich, 1971).

### Clonogenic fraction after radiation or hyperthermia

At 24 h after seeding on a 25 $cm^2$ tissue culture flask (4 or $3 \times 10^5$ cells/flask), the exponentially growing cells were exposed to radiation or hyperthermia. The cells were exposed to various doses of radiation with $^{60}Co$ source at approximately 1 Gy $min^{-1}$. For hyperthermia, the cells were heated by immersing the flasks in a circulating water bath at 42°C for the indicated time. After these treatments, the cells were trypsinized, diluted at different densities and plated in 60 mm tissue culture dishes, in triplicate. After culture for 10 days, the resultant colonies were fixed, stained with crystal violet and colonies of over 50 cells were counted. Clonogenic fraction was calculated as: survival fraction = number of colonies/number of plated cells × PE (plating efficiency), where PE is colony-forming efficiency without any treatment. All experiments were repeated three times, independently.

### MTT assay for chemosensitivity

Cytotoxic agents used in this study were doxorubicin, mitomycin C, 5-fluorouracil (Kyowa Hakko Kogyo Co Ltd, Tokyo, Japan) and paclitaxel (Bristol-Myers Squibb KK, Tokyo, Japan). HCT116 ($2.5 \times 10^3$ per well) and DLD1 cells ($5 \times 10^3$ per well) were incubated with various concentrations of each chemotherapeutic agent in 96-well plates. After 72 h of incubation, both 20 μl of 0.4% 3-(4,5-dimethylthiazol-2-yl)-2,5-diphenyl-tetrazolium bromide (MTT; Sigma) and 20 μl of succinic acid were added to each well. After a further 3 h of incubation, spectrometric absorbance at 540 nm was measured using a microplate-reader (Tosoh Co Ltd, Tokyo, Japan) with the absorbance set at 620 nm as a background. The growth inhibition rates of the cells were calculated as follows: percentage of growth inhibition = (B – A)/B × 100 (%), in which A and B are the mean absorbance value of cells cultured with and without a drug, respectively. Chemosensitivity of the Mn-SOD antisense clones was indicated as a relative ratio of growth inhibition compared to that of the vector control clone treated using the same conditions.

### Cytofluorometric analysis of mitochondrial transmembrane potential and DNA content

Mitochondrial transmembrane potential ($\Delta\Psi_m$) was measured by RHODAMINE 123 (Rh 123; Molecular Probes Inc, Eugene, OR, USA) staining (Shimizu et al, 1996). In brief, the culture cells were trypsinized at 24 h after radiation and resuspended in the complete medium containing Rh 123 (10 μM) at 37°C for 15 min. After washing twice with phosphate-buffered saline (PBS), the cells were further resuspended in PBS with 10 μM propidium iodide (PI; Molecular Probes Inc) for 10 min and analysed using flow cytometry (Coulter Corp, Hialeah, FL, USA). PI positive cells

© 2000 Cancer Research Campaign were excluded for determination of $\Delta\Psi_m$ of viable cells. Control experiments were done in the presence of 60 μM carbonyl cyanide m-chlorophenylhydrazone (CCCP; Sigma) for 30 min.

To measure the percentage of apoptotic nuclei after radiation, DNA contents were analysed based on PI staining. Cells collected at the indicated time after radiation were fixed with 70% ethanol for 1–2 days and rehydrated in buffer consisting of 50 mM $Na_2HPO_4$ (9 parts), 25 mM citric acid (1 part), and 0.1% Triton-X100 (Hotz et al, 1994). An aliquot of the cells was then stained with 2 μg $ml^{-1}$ PI, containing 50 U $ml^{-1}$ RNase A, for 30 min before measuring the fluorescence.

### DNA fragmentation assay

To determine oligonucleosomal DNA fragmentation, nuclear DNA (20 μg) obtained from the cells lysed by proteinase K and RNase A was subjected to 2% agarose gel electrophoresis, followed by ethidium bromide staining (Zamzami et al, 1995).

### Statistical analysis

Two-tailed Student's *t*-test was used for statistical analysis. $P < 0.05$ was considered to have statistical significance.

### RESULTS

### Establishment of stable transfectants of HCT116 and DLD1 colon cancer cell lines expressing Mn-SOD antisense RNA

Transfection to HCT116 and DLD1 colon cancer cells with the Mn-SOD antisense RNA expression plasmid or the control empty plasmid was done and neomycin-resistant cells were cloned by dilution cloning. The Mn-SOD protein levels in the selected clones were assayed by immunoblotting analysis, using anti-Mn-SOD polyclonal antibodies (kindly provided from Professor Naoyuki Taniguchi, Osaka University School of Medicine). Finally two stable clones with decreased Mn-SOD protein levels were established from HCT116 and DLD1 and were designated H-A16 and H-A22, and D-A10 and D-A22, respectively. The Mn-SOD activity of these stable antisense clones was reduced to 27–59%, compared to the respective vector control clones (Table 1). Northern blot analysis shows decreased expression levels of 4 kb Mn-SOD mRNA in antisense-transfected cell lines and no considerable changes in Mn-SOD mRNA expression levels between parental and vector-transfected cell lines, indicating the changes of Mn-SOD activities observed in the antisense clones are not due to drug selection (Figure 1). There was no significant difference of the Mn-SOD activity between the parental cells and vector controls of both cell lines. No significant difference of Cu,Zn-SOD activity between the vector controls and the Mn-SOD antisense clones was detected by SOD native gel assay (data not shown).

**Table 1** Relative Mn-SOD activity of stable Mn-SOD antisense clones

| Clones[a] | Relative Mn-SOD activity (±SD)[b] compared with the vector control |
|---|---|
| H-A16 | 0.59 ± 0.09 |
| H-A22 | 0.33 ± 0.02 |
| D-A10 | 0.57 ± 0.24 |
| D-A22 | 0.27 ± 0.09 |

[a]Two stable antisense clones from HCT116 and DLD1 were designated H-A16, H-A22 and D-A10, D-A22, respectively; [b]Mn-SOD activity was evaluated using the nitroblue tetrazolium (NBT) method (Oberley and Spitz, 1985), in three independent experiments

### Effects of suppression of Mn-SOD on sensitivity to radiation, hyperthermia and anticancer drugs

The sensitivity to various anti-cancer treatments was compared between the Mn-SOD antisense and the vector control clones established from HCT116 and DLD1 colon cancer cell lines. Figure 2A shows the clonogenic survival of those clones after exposure to 3 Gy irradiation. Both of two HCT116 antisense clones (H-As) showed significantly increased sensitivity to radiation ($P < 0.05$ in H-A16 and $P < 0.01$ in H-A22). The degree of the increase of sensitivity in H-As was comparable to that of suppression of those for Mn-SOD activity (Table 1). Neither DLD1 antisense clone (D-As) D-A10 nor D-A22 showed any significant changes in sensitivity. Increased sensitivity to radiation of H-A16 and H-A22 was radiation dose-dependent (Figure 2B).

Next, the sensitivity to hyperthermia in the H-As and D-As was evaluated. Both of the H-As showed significantly enhanced susceptibility to hyperthermia at 42°C as compared to the vector control, in a dose-dependent manner (Figure 3A), while no changes were observed in the survival rate in the D-As (data not shown). The extent of the decrease of clonogenic survival in hyperthermia also depended on the Mn-SOD activity. Sensitivity of the Mn-SOD antisense clones to some chemotherapeutic agents was also measured by MTT assay. Doxorubicin, whose cyotoxicity is considered to be partly due to $O_2^{\cdot-}$ generated, showed more cytotoxicity in the H-As than the HCT116 vector control clone but not in the D-As (Figure 3B). Augmentation of cytotoxicity of doxorubicin was achieved more significantly in low-dose administration. None of the Mn-SOD antisense clones demonstrated any enhancement in sensitivity to any other cytotoxic agents, including 5-fluorouracil, mitomycin C and paclitaxel (data not shown).

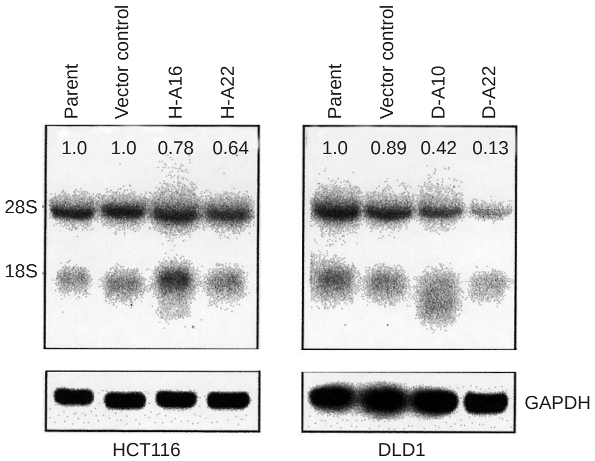

**Figure 1** Northern blot analysis is performed with $^{32}$P-labelled random primed probe using PCR-amplified Mn-SOD cDNA and glyceraldehyde-3-phosphate dehydrogenase (GAPDH) cDNA as templates. Relative intensity of each 4 kb Mn-SOD mRNA in antisense clones to parental cells, which is normalized by that of each GAPDH mRNA intensity, is indicated

 © 2000 Cancer Research Campaign

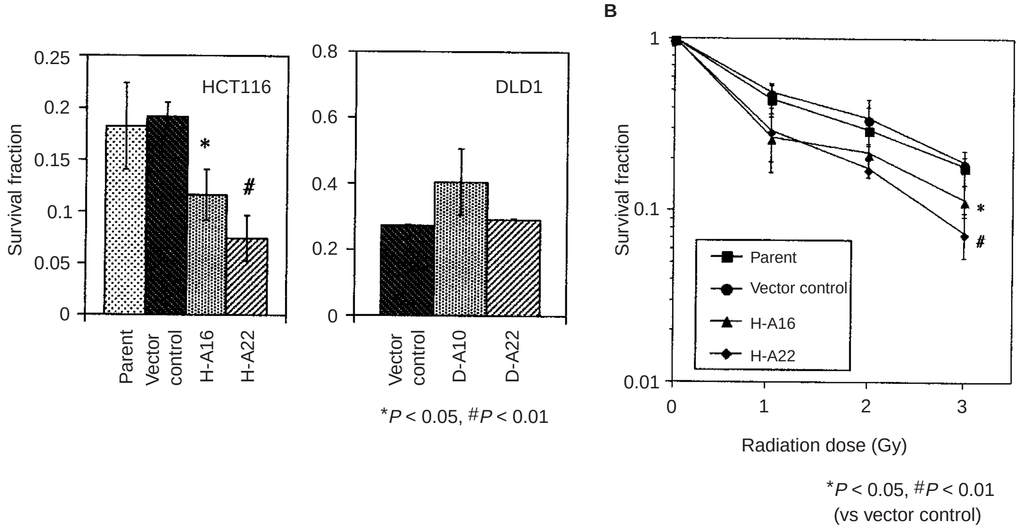

**Figure 2** (**A**) Mn-SOD antisense clones of HCT116 and DLD1 were exposed to 3 Gy irradiation and surviving fractions were examined by colony formation assay. HCT116 antisense clones exhibited higher sensitivity than did the vector control, whereas no significant difference of survival fraction was seen in DLD1 antisense clones. (**B**) Survival fractions of HCT116 and its stable transfectants and the indicated dose of irradiation are shown and the bars are the SEM. Susceptibility of HCT116 antisense clones to radiation was dose-dependent. All data indicate a mean survival fraction and the bars represent the SEM in three independent experiments

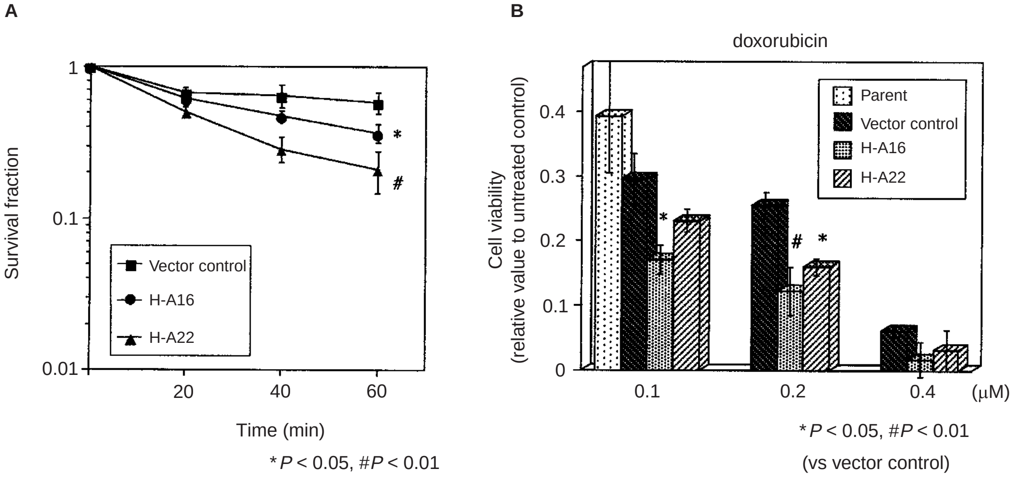

**Figure 3** Sensitivities of Mn-SOD antisense transfectants of HCT116 to hyperthermia (**A**) and doxorubicin (**B**) were evaluated by colony formation assay and MTT assay, respectively. The mean survival fraction of the indicated duration of hyperthermia was examined in three independent experiments. Cell viability after treatment of the indicated amounts of doxorubicin was expressed as a relative value to the untreated control. The bars indicate the SEM

### Suppression of Mn-SOD enhances apoptosis induced by radiation in HCT116 antisense clones

To examine the mechanism of enhanced radiosensitivity in the H-As, we analysed the extent of mitochondrial permeability transition (PT) induced by radiation in H-As. After 24 h of 3 Gy irradiation, the magnitudes of $\Delta\Psi_m$ reduction in H-A16 and H-A22 were greater than that in the vector control and they exceeded the extent of $\Delta\Psi_m$ decrease after exposure of the vector control to an uncoupler CCCP (60 μM, 30 min) (Figure 4A, B). Since diminution of $\Delta\Psi_m$ is thought to be an early step of programmed lymphocyte death (Zamzami et al, 1995), induction of apoptosis in those clones was then examined by DNA fragmentation. Flow cytometric analysis revealed that the sub-G1 fraction, which is the DNA subdiploidy population in the H-As, increased as compared to the parental cell and the vector control after 10 days of irradiation (Figure 5A), suggesting that apoptosis was indeed induced. The percentage of subdiploidy in the H-As was more than twice of the vector control under irradiation (Figure 5B) and this was the case with hyperthermia (Figure 5C). The significant increase of nuclear subdiploidy population after irradiation was detected later than 3 days after irradiation, which shows that the mitochondrial PT preceded to apoptosis (data not shown). DNA fragmentation induced by irradiation was further confirmed by existence of the DNA ladder in the H-As (Figure 5D).

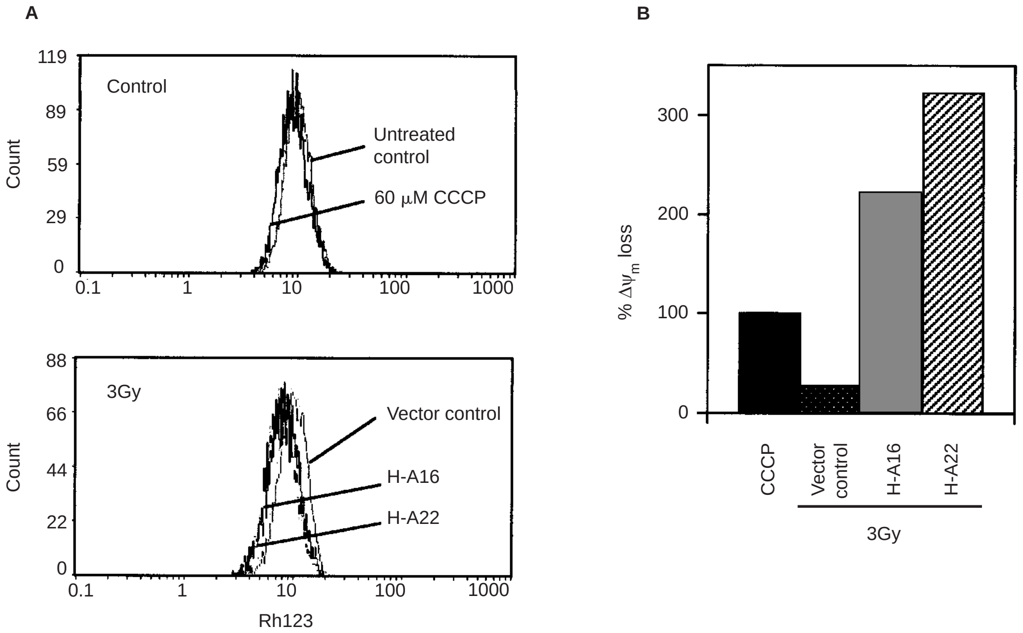

**Figure 4** $\Delta\Psi_m$ was examined by Rh 123-based staining. After excluding PI-positive cells, $\Delta\Psi_m$ of the viable cells was counted using a flow cytometer. Three independent experiments were done to evaluate the changes of $\Delta\Psi_m$. (**A**) Control experiments were performed by exposing the vector control to CCCP (60 μM, 30 min) and reduction of $\Delta\Psi_m$ in HCT116 antisense clones after 24 h of irradiation was assessed. (**B**) The magnitude of $\Delta\Psi_m$ loss was evaluated by comparing the values of the transfectants and CCCP (100%)

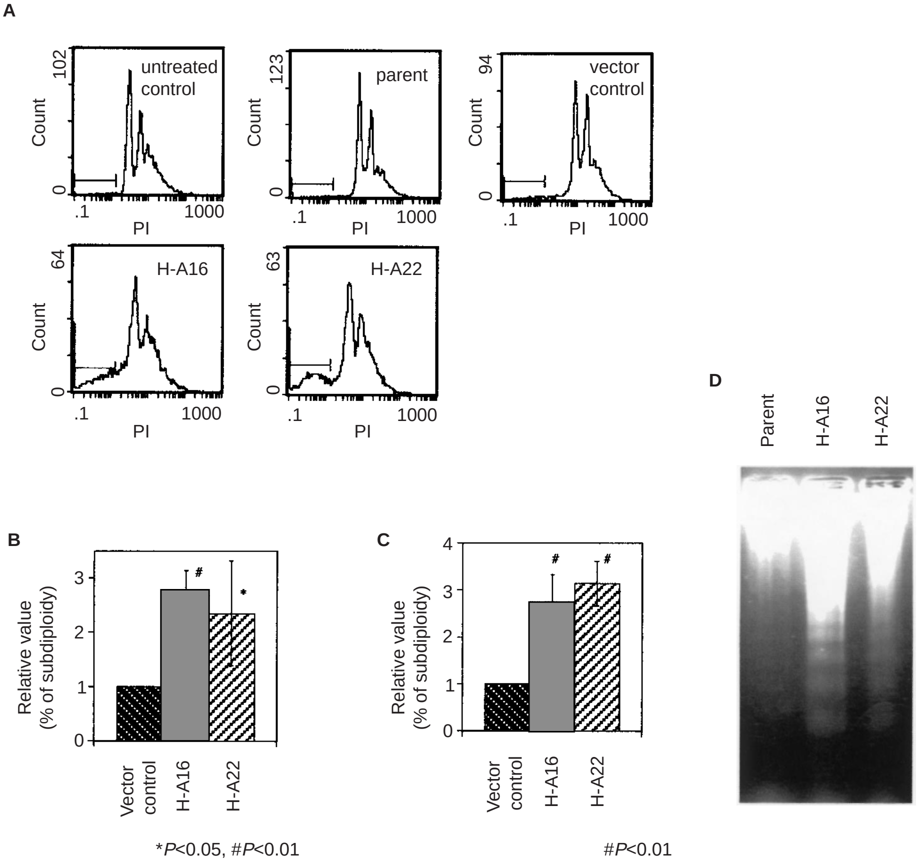

**Figure 5** To detect DNA fragmentation, ethanol-fixed cells were stained and a fraction of DNA subdiploidy was analysed with flow cytometry. (**A**) Increase of sub-G1 fraction was seen in HCT116 antisense clones after 10 days of 3 Gy irradiation, as compared to the parent and the vector control. (**B**) Population of sub-G1 fraction was compared as a relative value to the vector control. (**C**) Hyperthermia also induced more than twice as many DNA subdiploidy populations as that of the vector control. Data were obtained from three independent experiments and the bars indicate standard deviation. (**D**) Cellular DNA (20 μg) extracted from the cells at 10 days after exposure to 3 Gy irradiation was electrophoresed through a 2.0% agarose gel

*© 2000 Cancer Research Campaign*

### DISCUSSION

Resistance to various anticancer therapies such as chemotherapy, radiotherapy and hyperthermia still remains one of the major problems in the treatment of subjects with colon cancers. Therefore, efforts to clarify mechanisms involved in the resistance and to develop effective strategies to overcome the resistance are needed. Apoptosis is induced as the cellular response to the diverse anticancer therapies, and cancer cells which have acquired the resistance to apoptosis seem to have a growth advantage (Fisher, 1994; Graeber et al, 1996). Lowering the apoptotic threshold in the resistant cancer cells may be a promising strategy to enhance the effects of anti-cancer treatments.

Radiation, some chemo-immunotherapies and hyperthermia exert anti-tumour effects by generating excessive amounts of noxious ROS over the capacity of the endogenous antioxidant system (Sinha and Mimnaugh, 1990; Yoshikawa et al, 1993; Wallace, 1999). The forced expression of antioxidant Mn-SOD, which scavenges the apoptosis-inducible ROS in the mitochondria, makes cancer cells resistant to various anticancer treatments (Wong et al, 1989; Hirose et al, 1993; Li and Oberley, 1997). Mitochondria have recently been shown to play an important role in apoptosis (Susin et al, 1998; Green and Reed, 1998) and ROS, most of which are generated in the mitochondria, are involved in mitochondria-related apoptosis (Buttke and Sandstrom, 1994; Wallace, 1999). Thus, acceleration of ROS production in the mitochondria by suppression of Mn-SOD might lead to induction of apoptosis and aid in overcoming the resistance to anti-cancer therapies. We found that suppression of Mn-SOD, which would result in increase of the total amount of $O_2^{\cdot-}$ in the mitochondria, augmented apoptosis in colon cancer cells exposed to some treatment modalities which are thought to induce $O_2^{\cdot-}$ (Sinha and Mimnaugh, 1990; Yoshikawa et al, 1993; Wallace, 1999).

ROS can induce apoptosis by evoking mitochondrial PT (Susin et al, 1998) or by direct activation of caspase 3-like proteases (Higuchi et al, 1998). Furthermore, activated caspases are also shown to target the mitochondrial PT pore to open it (Marzo et al, 1998). Initiation of PT causes a reduction in $\Delta\Psi_m$, which is an irreversible step of programmed cell death (Zamzami et al, 1995), the result being release of apoptosis-inducing factor (AIF), cytochrome c and caspase zymogens to the cytosol from the mitochondrial inner-membrane space, after which AIF and downstream executors are activated to lead to apoptosis (Marzo et al, 1998; Susin et al, 1998; Green and Reed, 1998; Susin et al, 1999*a*; 1999*b*). Recently, Fujimura et al reported that Mn-SOD inhibits release of cytochrome c to the cytosol and reduces apoptosis after permanent focal cerebral ischaemia (Fujimura et al, 1999). In the Mn-SOD antisense clones of HCT116 colon cancer cells, the increased $O_2^{\cdot-}$ or its metabolites caused by suppression of Mn-SOD, may induce the mitochondrial PT pore, then cytochrome c and AIF released by PT induction may execute the signals to apoptosis. Indeed, PT induction preceded the DNA fragmentation in those antisense clones (data not shown). Furthermore, $O_2^{\cdot-}$ can also cause oxidative DNA damage by leaching iron from storage proteins and enzymatic (4Fe-4S) clusters (Keyer and Imlay, 1996; Fridovich, 1997); released iron catalyses the formation of the most reactive hydroxyl-radicals and damaged DNA could activate apoptosis-inducing signalling molecules such as p53.

The tumour suppressor gene p53 is known to make cancer cells susceptible to diverse anti-cancer treatments (Lowe at al, 1993; Muschel et al, 1998). p53 is activated in response to DNA damage and stimulates the transcription of proapoptotic genes such as Bax to execute apoptosis in affected cells (Kastan et al, 1991; Tishler et al, 1993; Miyashita and Reed, 1995). Furthermore, it has been shown that p53 itself induces apoptosis by generating ROS and subsequently causing oxidative mitochondrial impairment (Johnson et al, 1996; Polyak et al, 1997). We observed changes in sensitivity to various treatments in p53 wild-type colon cancer cell with reduced Mn-SOD activity but not in p53 mutated-type, suggesting the possibility that suppression of Mn-SOD in cancer cells under normal p53 functions may augment the p53-dependent apoptosis. In this study, however, we evaluated only two colon cancer cell lines, which have different backgrounds including DNA repair function and response to chemotherapeutic agents. Therefore, for our results to be more conclusive, further experiments would be required, i.e. either analyses of more colorectal cancer cells or co-transfection of wild-type p53 to the Mn-SOD antisense p53-mutant DLD1 cells and subsequent assessment of therapy resistance.

It was reported that Mn-SOD expression is reduced in cancer cells (Oberley and Oberley, 1997). However, recent studies have shown that some cancers such as central nervous system tumours, colorectal cancer and mesothelioma highly express Mn-SOD (Cobbs et al, 1996; Kinnula et al, 1996; Landriscina et al, 1996; Janssen et al, 1998). We also noted the higher expression of Mn-SOD mRNA in gastric and colorectal cancers than that in the paired mucosa. As Mn-SOD can be a resistant factor to radiation, hyperthermia and some anti-cancer drugs (Kinnula et al, 1996; Landriscina et al, 1996; Janssen et al, 1998), the high expression of Mn-SOD in the colon cancers may possibly contribute to resistance to those treatments. Our results indicate that suppression of Mn-SOD renders some colon cancer cells susceptible to those anti-cancer treatment modalities by inducing apoptosis. Thus, Mn-SOD could possibly be a target for enhancing effects of radiation, hyperthermia and doxorubicin in the p53 wild-type colon cancer.

### ACKNOWLEDGEMENTS

We thank Professor Naoyuki Taniguchi, Department of Biochemistry, Osaka University School of Medicine, Japan for providing anti-Mn-SOD antibodies and Professor Naoki Watanabe, Department of Laboratory Diagnosis, Sapporo Medical University, School of Medicine, Japan for helpful technical advice. This work was supported in part by a grant for the Second Term Comprehensive 10-year Strategy for Cancer Control from the Ministry of Health and Welfare, Japan. S Kuninaka is a recipient of a Research Resident Fellowship from the Foundation for Promotion of Cancer Research in Japan.

### REFERENCES

Beauchamp C and Fridovich I (1971) Superoxide dismutase: improved assays and an assay applicable to acrylamide gels. *Anal Biochem* **44**: 276–287

Buttke TM and Sandstrom PA (1994) Oxidative stress as a mediator of apoptosis. *Immunol Today* **15**: 7–10

Church SL, Grant JW, Ridnour LA, Oberley LW, Swanson PE, Meltzer P S and Trent JM (1993) Increased manganese superoxide dismutase expression suppresses the malignant phenotype of human melanoma cells. *Proc Natl Acad Sci USA* **90**: 3113–3117

Cobbs CS, Levi DS, Aldape K and Israel MA (1996) Manganese superoxide dismutase expression in human central nervous system tumours. *Cancer Res* **56**: 3192–3195

Fisher DE (1994) Apoptosis in cancer therapy: crossing the threshold. *Cell* **73**: 539–542

Fridovich I (1997) Superoxide anion radical ($O_2^{\cdot-}$), superoxide dismutases, and related matters. *J Biol Chem* **272**: 18515–18517

Fujimura M, Morita-Fujimura Y, Kawase M, Copin, JC, Calagui B, Epstein CJ and Chan PH (1999) Manganese superoxide dismutase mediates the early release of mitochondrial cytochrome c and subsequent DNA fragmentation after permanent focal cerebral ischemia in mice. *J Neurosci* **19**: 3412–3422

Graeber TG, Osmanian C, Jacks T, Housman DE, Koch CJ, Lowe SW and Giaccia AJ (1996) Hypoxia-mediated selection of cells with diminished apoptotic potential in solid tumours. *Nature* **379**: 88–91

Green DR and Reed JC (1998) Mitochondria and apoptosis. *Science* **281**: 1309–1312

Higuchi M, Honda T, Proske RJ and Yeh ETH (1998) Regulation of reactive oxygen species-induced apoptosis and necrosis by caspase 3-like proteases. *Oncogene* **17**: 2753–2760

Hirose K, Longo DL, Oppenheim JJ and Matsushima K (1993) Overexpression of mitochondrial manganese superoxide dismutase promotes the survival of tumour cells exposed to interleukin-1, tumour necrosis factor, selected anticancer drugs, and ionising radiation. *FASEB J* **7**: 361–368

Hotz HA, Gong J, Traganos F and Darzynkiewicz Z (1994) Flow cytometric detection of apoptosis: comparison of the assay of in situ DNA degradation and chromatin changes. *Cytometry* **15**: 237–244

Janssen YMW, Houten BV, Borm PJA and Mossman BT (1993) Cell and tissue responses to oxidative damage. *Lab Invest* **69**: 261–274

Janssen AML, Bosman CB, Sier CFM, Griffioen G, Kubben FJGM, Lamers CBHW, van Krieken JHJM, van de Velde CJH and Verspaget HW (1998) Superoxide dismutases in relation to the overall survival of colorectal cancer patients. *Br J Cancer* **78**: 1051–1057

Johnson TM, Yu Z-X, Ferrans VJ, Lowenstein RA and Finkel T (1996) Reactive oxygen species are downstream mediators of p53-dependent apoptosis. *Proc Natl Acad Sci USA* **93**: 11848–11852

Kastan MB, Onyekwere O, Sidransky D, Vogelstein B and Craig RW (1991) Participation of p53 protein in the cellular response to DNA damage. *Cancer Res* **51**: 6304–6311

Keyer K, Imlay JA (1996) Superoxide accelerates DNA damage by elevating free-iron levels. *Proc Natl Acad Sci USA* **93**: 13635–13640

Kinnula VL, Pietarinen-Runtti P, Raivio K, Kahlos K, Pelin K, Mattson K and Linnainmaa K (1996) Manganese superoxide dismutase in human pleural mesothelioma cell lines. *Free Radical Biol Med* **21**: 527–532

Landriscina M, Remiddi F, Ria F, Palazzotti B, De Leo ME, Iacoangeil M, Rosselli R, Scerrati M and Galeotti T (1996) The level of MnSOD is directly correlated with grade of brain tumours of neuroepithelial origin. *Br J Cancer* **74**: 1877–1885

Li JJ and Oberley LW (1997) Overexpression of manganese-containing superoxide dismutase confers resistance to the cytotoxicity of tumor necrosis factor α and/or hyperthermia. *Cancer Res* **57**: 1991–1998

Li JJ, Oberley LW, St. Clair DK, Ridnour LA and Oberley TD (1995) Phenotypic changes induced in human breast cancer cells by overexpression of manganese-containing superoxide dismutase. *Oncogene* **10**: 1989–2000

Lowe SW, Ruley HE, Jacks T and Housman DE (1993) p53-dependent apoptosis modulates the cytotoxicity of anticancer agents. *Cell* **74**: 957–967

Marzo I, Brenner C, Zamzami N, Susin SA, Beutner G, Brdiczka D, Remy R, Xie Z-H, Reed JC and Kroemer G (1998) The permeability transition pore complex: a target for apoptosis regulation by caspases and bcl-2-related proteins. *J Exp Med* **187**: 1261–1271

Miyashita T and Reed JC (1995) Tumor suppresser p53 is a direct transcriptional activator of the human bax gene. *Cell* **80**: 293–299

Muschel RJ, Soto DE, McKenna WG and Bernhard EJ (1998) Radiosensitization and apoptosis. *Oncogene* **17**: 3359–3363

Oberley LW and Buettner GR (1979) Role of superoxide in cancer: a review. *Cancer Res* **39**: 1141–1149

Oberley TD and Oberley LW (1997) Antioxidant enzyme levels in cancer. *Histol Histopathol* **12**: 525–535

Oberley LW and Spitz DR (1985) Nitroblue tetrazolium. In: *CRC Handbook of Methods for Oxygen Radical Research*, Greenwald RA (ed), pp 217–225. CRC Press Inc: Boca Raton, FL

Polyak K, Xia Y, Zweier JL, Kinzler KW and Vogelstein B (1997) A model for p53-induced apoptosis. *Nature* **389**: 300–305

Shimizu S, Eguchi Y, Kamiike W, Waguri S, Uchiyama Y, Matsuda H and Tsujimoto Y (1996) Bcl-2 blocks loss of mitochondrial membrane potential while ICE inhibitors act at a different step during inhibition of death induced by respiratory chain inhibitors. *Oncogene* **13**: 21–29

Sinha BK and Mimnaugh ED (1990) Free radicals and anticancer drug resistance: oxygen free radicals in the mechanisms of drug cytotoxicity and resistance by certain tumours. *Free Radical Biol Med* **8**: 567–581

Susin SA, Zamzami N and Kroemer G (1998) Mitochondria as regulators of apoptosis: doubt no more. *Biochim Biophys Acta* **1366**: 151–165

Susin SA, Lorenzo HK, Zamzami N, Marzo I, Snow BE, Brothers GM, Mangion J, Jacotot E, Costantini P, Loeffler M, Larochette N, Goodlett DR, Aebersold R, Siderovski DP, Penninger JM and Kroemer G (1999*a*) Molecular characterization of mitochondrial apoptosis-inducing factor. *Nature* **397**: 441–446

Susin SA, Lorenzo HK, Zamzami N, Marzo I, Brenner C, Larochette N, Provost M-C, Alzari PM and Kroemer G (1999*b*) Mitochondrial release of caspase-2 and -9 during the apoptotic process. *J Exp Med* **189**: 381–393

Tishler RB, Calderwood SK, Coleman CN and Price BD (1993) Increases in sequence specific DNA binding by p53 following treatment with chemotherapeutic and DNA damaging agents. *Cancer Res* **53**: 2212–2216

Toh Y, Kuninaka S, Oshiro T, Ikeda Y, Nakashima H, Baba H, Kohnoe S, Okamura T, Mori M and Sugimachi K (2000) Overexpression of manganese superoxide dismutase mRNA may correlate with aggressiveness in gastric and colorectal adenocarcinomas. *Int J Oncol* **17**: 107–112

Yoshikawa T, Kokura S, Tainaka K, Itani K, Oyamada H, Kaneko T, Naito Y and Kondo M (1993) The role of active oxygen species and lipid peroxidation in the antitumor effect of hyperthermia. *Cancer Res* **53**: 2326–2329

Wallace DC (1999) Mitochondrial diseases in man and mouse. *Science* **283**: 1482–1488

Wong GHW, Elwell JH, Oberley LW and Goeddel DV (1989) Manganous superoxide dismutase is essential for cellular resistance to cytotoxicity of tumor necrosis factor. *Cell* **58**: 923–931

Zamzami N, Marchetti P, Castedo M, Zanin C, Vayssiere J-L, Petit PX and Kroemer G (1995) Reduction in mitochondrial potential constitutes an early irreversible step of programmed lymphocyte death in vivo. *J Exp Med* **181**: 1661–1672

Zhong W, Oberley LW, Oberley TD, Yan T, Domann FE and St Clair D K (1996) Inhibition of cell growth and sensitization to oxidative damage by overexpression of manganese superoxide dismutase in rat glioma cells. *Cell Growth Differ* **7**: 1175–1186

 *© 2000 Cancer Research Campaign*
